# Agricultural crop density and risk of childhood cancer in the midwestern United States: an ecologic study

**DOI:** 10.1186/s12940-015-0070-3

**Published:** 2015-10-15

**Authors:** Benjamin J. Booth, Mary H. Ward, Mary E. Turyk, Leslie T. Stayner

**Affiliations:** Division of Epidemiology and Biostatistics, School of Public Health, University of Illinois at Chicago, Chicago, IL USA; Occupational and Environmental Epidemiology Branch, Division of Cancer Epidemiology and Genetics, National Cancer Institute, National Institutes of Health, Rockville, MD USA

**Keywords:** Childhood cancer, Environmental epidemiology, Crop density, Pesticides

## Abstract

**Background:**

There is limited evidence for an association between agricultural pesticide exposure and certain types of childhood cancers. Numerous studies have evaluated exposure to pesticides and childhood cancer and found positive associations. However, few studies have examined the density of agricultural land use as a surrogate for residential exposure to agricultural pesticides and results are mixed. We examined the association of county level agricultural land use and the incidence of specific childhood cancers.

**Methods:**

We linked county-level agricultural census data (2002 and 2007) and cancer incidence data for children ages 0–4 diagnosed between 2004 and 2008 from cancer registries in six Midwestern states. Crop density (percent of county area that was harvested) was estimated for total agricultural land, barley, dry beans, corn, hay, oats, sorghum, soybeans, sugar beets, and wheat. Rate ratios and 95 % confidence intervals were estimated using generalized estimating equation Poisson regression models and were adjusted for race, sex, year of diagnosis, median household income, education, and population density.

**Results:**

We found statistically significant exposure-response relationships for dry beans and total leukemias (RR per 1 % increase in crop density = 1.09, 95 % CI = 1.03–1.14) and acute lymphoid leukemias (ALL) (RR = 1.10, 95 % CI = 1.04–1.16); oats and acute myeloid leukemias (AML) (RR = 2.03, 95 % CI = 1.25, 3.28); and sugar beets and total leukemias (RR = 1.11, 95 % CI = 1.04, 1.19) and ALL (RR = 1.11, 95 % CI = 1.02, 1.21). State-level analyses revealed some additional positive associations for total leukemia and CNS tumors and differences among states for several crop density-cancer associations. However, some of these analyses were limited by low crop prevalence and low cancer incidence.

**Conclusions:**

Publicly available data sources not originally intended to be used for health research can be useful for generating hypotheses about environmental exposures and health outcomes. The associations observed in this study need to be confirmed by analytic epidemiologic studies using individual level exposure data and accounting for potential confounders that could not be taken into account in this ecologic study.

**Electronic supplementary material:**

The online version of this article (doi:10.1186/s12940-015-0070-3) contains supplementary material, which is available to authorized users.

## Introduction

There is limited epidemiologic evidence that exposure to agricultural pesticides, mainly through parental occupational exposures, is associated with an increased risks of childhood leukemia and childhood central nervous system (CNS) and peripheral nervous system (PNS) cancers [1–4]. A few studies have examined crop density (i.e. the percentage of land planted in crops) around the birth or childhood residence as a surrogate for environmental exposure to agricultural pesticides and risk of these childhood cancers [5–7]. The use of crop density as an exposure metric is supported by studies examining agricultural pesticide levels in house dust and the density of crops [8], proximity from farmland [9–11], or agricultural pesticide use around the home [12]. For example, in Iowa, increased density of crops surrounding the home significantly increased the likelihood of detecting herbicides in the home [8]. In California, residences with higher density of specific agricultural pesticides within 1250 m of the home had significantly higher concentrations of the pesticide within the home compared to homes without use of the pesticide within 1250 m [12]. Furthermore, concentrations of specific herbicides in house dust have been associated with increased risk of childhood ALL [13].

Two studies used county level data from the U.S. Census of Agriculture to examine crop density and childhood cancer incidence [5, 6]. Walker et al. [5] found positive trends for CNS and brain cancer in Texas with increasing density of total cropland within the county of residence at birth, but did not examine associations with specific crops. In a study in 25 states, Carozza et al. [6] found significant positive associations between total crop density within the county of residence at diagnosis and leukemia and brain and other CNS cancers. Additional positive associations were observed between AML and soybean density; neuroblastomas and corn and soybean density; and primitive neuroectodermal tumors and oat density.

The current investigation used county-level estimates of crop density for total cropland and nine crop types from the 2002 and 2007 Census of Agriculture from six Midwestern states to assess the relationship with childhood leukemia, CNS and PNS cancer incidence rates. The impetus for this research was to demonstrate the utility of using publicly available environmental data in combination with existing health outcome data to explore environmental exposures and the risk of childhood cancers. In addition, this research was performed to provide leads to environmental causes of childhood cancer.

## Materials and methods

### Cancer incidence and population data

We obtained incidence data for leukemias, CNS and miscellaneous intracranial and intraspinal neoplasms, and neuroblastoma and other peripheral nervous cell tumors from 2004 through 2008 for children under the age of five from state cancer registries in Illinois, Indiana, Ohio, Michigan, and Missouri; data for Iowa were obtained through the Surveillance, Epidemiology, and End-Results (SEER) program’s SEER*Stat software version 8.1.5 [14]. International Classification of Childhood Cancer, third edition (ICCC-3) site codes were used to categorize childhood cancer records into total leukemias (011–015), ALL (011), AML (012), all brain and other CNS and PNS cancers (031–042), CNS and miscellaneous neoplasms (031–036), and neuroblastomas and other PNS cancers (041–042). Residence at the time of cancer diagnosis was used to compute county-level cancer incidence rates (as described below).

County-level intercensal estimates of the county populations stratified by age group, race, and sex from the US Census Bureau were used as the denominators for estimating rates [15]. Median household income and educational attainment data were obtained from the 2005 through 2009 American Community Survey [16]. Educational attainment was represented by the percent of the population 25 years of age and over with at least a bachelor’s degree.

### County acreage and percent acres in specific crops data

Data on harvested acres of total cropland and by type of crop harvested for each county in the six states were obtained from the 2002 and 2007 U.S. Department of Agriculture (USDA) National Agricultural Statistics Service (NASS) Census of Agriculture [17, 18]. Data from annual NASS surveys were considered, but were found to be less comprehensive when compared to the Census of Agriculture data. We averaged crop acreage from the 2002 and 2007 censuses, a time period that preceded or overlapped with the case diagnosis period (2004–2008).

Estimates of crop acreage were available for all states for barley, corn, hay, oats, sorghum, soybeans, and wheat. In addition, harvested acreage of dry beans was available for all states except for Illinois, and acreage of sugar beets was available for Michigan and Ohio. The Census of Agriculture withheld precise crop acreage for a county when there was a chance of disclosing data for individual farms (generally less than four), and instead reported the number of farms that were in categories of crop acreage harvested. These categories were 1.0 to 24.9 acres, 25.0 to 99.9 acres, 100 to 249 acres, 250 to 499 acres, 500 to 999 acres, and 1000 or more acres. For counties with acreage reported in categories, crop acreage was computed by multiplying the midpoint of each acreage category by the number of farms in the category and summing across all categories of farms. Total land area in square kilometers for each county was obtained from the U.S. Census Bureau 2013 Tiger/Line File [19]. The percentage of total cropland and specific crop types was created by dividing the harvested crop acres by the county land area in acres. Population density was computed by dividing the total population in each county by the total land area in square kilometers in each county.

### Statistical analysis

Three Poisson-based regression models were used to determine the impact of including spatially varying information and to assess the validity of independence assumptions [20]. Models were fitted separately for each type of crop density. First, Poisson regression was used to generate models without any consideration of spatial or temporal clustering of the data. Second, generalized estimating equation (GEE) Poisson regression models were fitted to account for group level clustering by county. County was treated as a repeated measure in these models. Finally, distance decay random effects Poisson regression models were used to account for the geographically varying components of the data such as crop locations [21]. These models used an adjacency matrix that assigned counties a value of 0 or 1 to indicate adjacency. County was treated as a random effect in these models. Results were consistent among the three modeling approaches; therefore, only the GEE Poisson models are presented.

Rate ratios (RR) and 95 % confidence intervals (CI) were adjusted for year, race, sex, county median household income, educational attainment, and population density. Additionally, all models were restricted to include only counties with populations of less than 300,000 people to reduce the potential for confounding by inclusion of urban areas, which may have different risk factors for childhood cancers than rural areas. Our analyses included 551 counties in the six states.

We had no reason to expect the same relationships between each crop and cancer type, so we explored multiple exposure definitions to account for both linear and non-linear associations. Crop density was modeled as binary, quartile, and continuous variables for corn, hay, soybeans, wheat, and total agricultural land. Binary categories were defined as less than or equal to the median and above the median. Quartiles were based on crop density cut points that gave an approximately equal number of counties in each group. Barley, dry beans, oats, sorghum, and sugar beets had limited distributions and many counties had no acreage of these crops. Therefore, categorical variables were created with zero acreage as the reference group and non-zero values were categorized into two groups based on the median acreage. Binary variables were modeled as any versus no acres harvested. Wald tests for a log linear exposure-response trend were based on the continuous estimates of crop density. Restricted cubic splines were used to further assess the shape of the crop density-cancer outcomes relationships and to test for non-linear relationships.

SAS Version 9.2 (SAS Institute Inc., Cary, NC, USA) was used to generate the Poisson regression and generalized estimating equation Poisson regression models. Stata version 13.0 (StataCorp, College Station, TX, USA) was used to perform restricted cubic spline analyses. The distance decay random effects Poisson regression models used the Geographic and Multi-level Models for Environmental Public Health Indicators and Tracking (GAMEPHIT) program in R Version 2.15.1 (R Foundation for Statistical Computing, Vienna, Austria) [21].

## Results

A total of 664 cases of leukemia (518 ALL and 94 AML) and 691 cases of CNS/PNS (315 CNS and 218 PNS) cancers among an average annual population of 1,639,649 children less than five years of age were included in the study (Table 1). By state, the number of cases of ranged from 59 to 158 for CNS and PNS cancers and from 62 to 152 for the leukemias. Incidence rates per 100,000 children under the age of five for total leukemia ranged from 37.7 to 42.8 and for total CNS and PNS cancers ranged from 31.4 to 51.9.

**Table 1:** Cases, rates^a^, and population at risk from six Midwestern states included in crop analysis, 2004–2008, excluding counties with populations >300,000

			Total Leukemia		ALL		AML		CNS/PNS		CNS		PNS	
	Number of counties	At-risk population^b^	Cases	Rate	Cases	Rate	Cases	Rate	Cases	Rate	Cases	Rate	Cases	Rate
Illinois	96	286,387	108	37.7	88	30.7	13	4.5	90	31.4	51	17.8	39	13.6
Indiana^c^	89	304,230	127	41.7	106	34.8	12	3.9	158	51.9	-	-	-	-
Iowa	98	162,469	62	38.2	51	31.4	10	6.2	59	36.3	39	24.0	20	12.3
Michigan	77	282,812	117	41.4	88	31.1	18	6.4	135	47.7	78	27.6	57	20.2
Missouri	111	228,951	98	42.8	71	31.0	20	8.7	94	41.1	48	21.0	46	20.1
Ohio	80	374,800	152	40.6	114	30.4	21	5.6	155	41.4	99	26.4	56	14.9
Total	551	1,639,649	664	40.5	518	31.6	94	5.7	691	42.1	315^a^	19.2	218^a^	13.3

^a^Crude rates per 100,000 children. ^b^Average annual population at risk, children 0–4 years of age. ^c^Separate CNS and PNS counts were not provided for Indiana. - Cases and rates not available

Based on the counties in our analyses, Iowa and Illinois had the highest median percentages of county agricultural land at 76.6 % and 72.0 %, respectively, while Michigan had the lowest median percentage of county agricultural land at 16.8 % (Table 2). Corn and soybeans were the most commonly harvested crops for all states except for Michigan, where hay had the highest median county crop density (2.9 %) and corn had the second highest density (2.0 %), and Missouri, where soybeans (9.7 %) and hay (9.2 %) had the highest densities. Barley, dry beans, oats, sorghum, and sugar beets had the lowest median percentages of crops across each state. Dry beans were grown in five of the six states and sugar beets in two of the six states (Michigan and Ohio), but median percentages were zero reflecting the small number of counties in which these crops were grown.

**Table 2:** Median (min, max) county crop density (percentage of total acreage) for six Midwestern states, excluding counties with populations >300,000

Crop	Illinois	Indiana	Iowa	Michigan	Missouri	Ohio	Total
Agricultural land	72.04 (16.80, 90.83)	59.24 (4.52, 96.11)	76.63 (40.89, 94.41)	16.79 (0.14, 73.97)	43.28 (5.91, 97.71)	44.57 (7.18, 95.20)	57.69 (0.14, 97.71)
Barley	0.00 (0.00, 0.05)	0.00 (0.00, 0.07)	0.00 (0.00, 0.12)	0.02 (0.00, 0.22)	0.00 (0.00, 0.10)	0.01 (0.00, 0.25)	0.00 (0.00, 0.25)
Beans (dry)	-	0.00 (0.00, 0.14)	0.00 (0.00, 0.14)	0.02 (0.00, 14.98)	0.00 (0.00, 0.08)	0.00 (0.00, 0.14)	0.00 (0.00, 14.98) ^a^
Corn	34.39 (2.15, 53.68)	26.31 (0.88, 51.12)	37.97 (7.02, 52.50)	2.01 (0.00, 28.44)	4.65 (0.00, 35.88)	11.29 (0.14, 31.11)	19.1 (0.00, 53.68)
Hay	1.61 (0.29, 7.51)	1.90 (0.39, 7.94)	2.63 (0.48, 11.33)	2.92 (0.05, 9.77)	9.23 (0.09, 22.71)	3.54 (0.85, 12.57)	2.84 (0.05, 22.71)
Oats	0.04 (0.00, 0.65)	0.02 (0.00, 0.60)	0.21 (0.01, 1.28)	0.14 (0.00, 0.82)	0.02 (0.00, 0.18)	0.09 (0.00, 2.18)	0.07 (0.00, 2.18)
Sorghum	0.03 (0.00, 2.72)	0.00 (0.00, 0.67)	0.00 (0.00, 0.25)	0.00 (0.00, 0.06)	0.13 (0.00, 3.27)	0.00 (0.00, 0.14)	0.00 (0.00, 3.27)
Soybeans	28.11 (2.88, 42.98)	24.12 (0.73, 44.62)	28.71 (6.23, 40.64)	0.44 (0.00, 24.15)	9.66 (0.00, 61.20)	16.10 (0.01, 47.99)	20.12 (0.00, 61.20)
Sugar beets	-	-	-	0.00 (0.00, 10.28)	-	0.00 (0.00, 0.32)	0.00 (0.00, 10.28) ^b^
Wheat	1.10 (0.05, 18.37)	0.94 (0.08, 11.98)	0.03 (0.00, 0.66)	0.37 (0.00, 8.55)	1.02 (0.00, 18.92)	2.25 (0.01, 12.42)	0.71 (0.00, 18.92)

^a^Excluding Illinois. ^b^Harvested in Michigan and Ohio only. - Crop not grown in this state. Agricultural land is total agricultural land including the nine crops under study

### Combined state analysis

In the analysis of all six states combined, we found no evidence of a significant linear exposure-response relationship between density of total cropland, barley, corn, hay, or soybeans and any of the cancer outcomes (Table 3). We observed significant associations for total leukemias and ALL with dry beans (total leukemias RR for a 1 % change = 1.09, 95 % CI = 1.03–1.14) and sugar beets (total leukemias RR 1 % = 1.10, 95 % CI = 1.04–1.16). Sugar beets were only grown in Michigan and Ohio in a small proportion of counties (27 for Michigan and 5 for Ohio) and our analyses were limited to these states. Counties with higher densities of dry beans and oats had significantly increased AML incidence. We also observed some inverse associations. Highest densities of oats, sorghum, and wheat were associated with significantly lower rates of total leukemia and/or ALL. We observed no significant associations between any of the crop density variables and CNS and PNS incidence rates.

**Table 3:** Estimated rate ratios (RRs)^a^ and 95 % confidence intervals (CI) of childhood cancers (0–4 years of age) associated with cropland density overall and by crop type in six Midwestern states, 2004-2008

Crop	Crop density (%)^b^	Total leukemia	ALL	AML	CNS/PNS	CNS^c^	PNS^c^
Agricultural	31.0–57.6	0.75 (0.63, 0.89)	0.80 (0.65, 0.98)	0.74 (0.45, 1.23)	1.04 (0.85, 1.28)	1.16 (0.85, 1.59)	1.00 (0.74, 1.34)
Land	57.7–73.9	0.89 (0.71, 1.12)	0.93 (0.71, 1.20)	0.64 (0.32, 1.30)	1.15 (0.89, 1.48)	1.16 (0.80, 1.69)	1.14 (0.76, 1.72)
	>73.9	1.01 (0.77, 1.31)	1.05 (0.78, 1.42)	0.64 (0.27, 1.53)	1.17 (0.87, 1.58)	1.27 (0.83, 1.94)	1.03 (0.64, 1.68)
	Continuous	1.00 (0.99, 1.00)	1.00 (0.99, 1.00)	1.00 (0.98, 1.01)	1.00 (0.99, 1.00)	1.00 (0.99, 1.01)	1.00 (0.99, 1.01)
	Median (>/≤)	1.05 (0.88, 1.25)	1.02 (0.83, 1.26)	0.88 (0.51, 1.52)	1.13 (0.93, 1.38)	1.09 (0.82, 1.46)	1.11 (0.78, 1.56)
Barley^d^	0.001–0.015	0.91 (0.75, 1.11)	0.91 (0.73, 1.15)	0.97 (0.57, 1.65)	0.96 (0.77, 1.20)	0.99 (0.68, 1.43)	0.96 (0.67, 1.38)
	>0.015	0.99 (0.80, 1.22)	0.94 (0.74, 1.19)	1.31 (0.75. 2.30)	0.90 (0.74, 1.09)	0.78 (0.57, 1.06)	1.20 (0.88, 1.65)
	Continuous	0.40 (0.04, 4.28)	0.11 (0.01, 2.90)	111.96 (0.94, 13334.27)	0.25 (0.02, 3.48)	0.08 (0.01, 10.67)	3.28 (0.09, 115.07)
	Any/None	0.92 (0.78, 1.07)	0.91 (0.76, 1.09)	1.04 (0.67, 1.61)	0.96 (0.81, 1.14)	0.93 (0.72, 1.22)	1.07 (0.81, 1.41)
Beans	0.002–0.036	0.88 (0.69, 1.13)	0.88 (0.65, 1.19)	0.99 (0.57, 1.74)	0.87 (0.68, 1.11)	0.81 (0.55, 1.21)	0.76 (0.53, 1.08)
(dry)^e,f^	>0.036	1.08 (0.88, 1.32)	0.99 (0.77, 1.26)	1.73 (1.07, 2.78)	0.96 (0.73, 1.26)	0.79 (0.53, 1.16)	1.09 (0.73, 1.62)
	Continuous	1.09 (1.03, 1.14)	1.10 (1.04, 1.16)	0.94 (0.75, 1.17)	1.03 (0.96, 1.11)	1.02 (0.91, 1.14)	1.05 (0.96, 1.16)
	Any/None	0.97 (0.80, 1.16)	0.93 (0.74, 1.16)	1.28 (0.85, 1.91)	0.91 (0.74, 1.12)	0.80 (0.57, 1.12)	0.91 (0.67, 1.24)
Corn	4.8–19.1	0.85 (0.70, 1.03)	0.85 (0.68, 1.06)	0.91 (0.51, 1.62)	1.03 (0.82, 1.30)	0.92 (0.64, 1.34)	1.24 (0.86, 1.78)
	19.2–32.6	0.75 (0.60, 0.95)	0.75 (0.58, 0.99)	0.86 (0.40, 1.84)	1.01 (0.77, 1.32)	0.93 (0.64, 1.36)	1.24 (0.81, 1.90)
	>32.6	1.11 (0.81, 1.51)	1.06 (0.75, 1.50)	1.01 (0.35, 2.91)	1.16 (0.80, 1.67)	1.02 (0.58, 1.78)	1.55 (0.81, 2.95)
	Continuous	1.00 (0.99, 1.01)	1.00 (0.99, 1.01)	0.99 (0.97, 1.02)	1.00 (0.99, 1.01)	1.00 (0.99, 1.01)	1.01 (0.99, 1.02)
	Median (>/≤)	0.89 (0.74, 1.06)	0.90 (0.73, 1.10)	0.84 (0.44, 1.58)	0.98 (0.80, 1.20)	0.98 (0.73, 1.33)	1.07 (0.78, 1.48)
Hay	1.6–2.8	0.87 (0.71, 1.08)	0.91 (0.72, 1.16)	0.71 (0.37, 1.38)	1.03 (0.81, 1.32)	1.16 (0.81, 1.65)	1.09 (0.70, 1.69)
	2.9–5.8	1.11 (0.91, 1.35)	1.08 (0.86, 1.37)	1.20 (0.63, 2.30)	1.09 (0.86, 1.38)	1.16 (0.82, 1.65)	1.02 (0.67, 1.54)
	>5.8	0.93 (0.70, 1.23)	0.95 (0.68, 1.31)	1.20 (0.59, 2.46)	0.92 (0.67, 1.26)	0.96 (0.61, 1.50)	1.04 (0.63, 1.74)
	Continuous	1.00 (0.98, 1.03)	1.00 (0.97, 1.04)	1.03 (0.97, 1.10)	1.00 (0.97, 1.03)	1.01 (0.96, 1.06)	0.98 (0.94, 1.02)
	Median (>/≤)	1.16 (0.99, 1.35)	1.11 (0.93, 1.33)	1.47 (0.92, 2.26)	1.03 (0.86, 1.23)	1.15 (0.88, 1.50)	0.84 (0.64, 1.12)
Oats	0.02–0.07	0.91 (0.74, 1.12)	0.90 (0.72, 1.13)	0.80 (0.44, 1.44)	1.03 (0.82, 1.30)	1.32 (0.89, 1.95)	0.87 (0.60, 1.28)
	0.08–0.18	0.79 (0.63, 0.99)	0.73 (0.56, 0.95)	0.97 (0.55, 1.72)	0.97 (0.77, 1.23)	1.11 (0.76, 1.60)	1.15 (0.75, 1.75)
	>0.18	0.91 (0.75, 1.11)	0.82 (0.65, 1.02)	1.34 (0.76, 2.38)	1.03 (0.80, 1.32)	1.10 (0.75, 1.60)	1.30 (0.86, 1.97)
	Continuous	0.89 (0.64, 1.22)	0.66 (0.44, 0.98)	2.03 (1.25, 3.28)	0.92 (0.67, 1.25)	0.90 (0.52, 1.55)	1.06 (0.67, 1.67)
	Median (>/≤)	0.88 (0.74, 1.05)	0.81 (0.67, 0.98)	1.24 (0.79, 1.96)	0.98 (0.82, 1.18)	0.90 (0.68, 1.20)	1.23 (0.89, 1.71)
Sorghum^g^	0.001–0.051	0.99 (0.82, 1.20)	0.96 (0.77, 1.18)	1.24 (0.75, 2.03)	1.06 (0.86, 1.29)	1.18 (0.88, 1.57)	0.85 (0.62, 1.16)
	>0.051	0.92 (0.70, 1.22)	0.90 (0.65, 1.24)	1.52 (0.74, 3.09)	1.02 (0.75, 1.39)	0.67 (0.39, 1.18)	1.01 (0.66, 1.54)
	Continuous	0.71 (0.53, 0.95)	0.79 (0.56, 1.12)	0.65 (0.34, 1.26)	0.89 (0.63, 1.24)	0.80 (0.41, 1.58)	0.89 (0.58, 1.38)
	Any/None	0.94 (0.80, 1.11)	0.93 (0.77, 1.12)	0.97 (0.61, 1.55)	0.99 (0.84, 1.18)	1.06 (0.82, 1.37)	0.81 (0.62, 1.06)
Soybeans	6.01–20.12	0.79 (0.66, 0.96)	0.80 (0.64, 0.99)	0.86 (0.52, 1.42)	1.01 (0.82, 1.25)	0.86 (0.62, 1.21)	1.23 (0.90, 1.66)
	20.13–29.99	0.83 (0.66, 1.04)	0.86 (0.66, 1.12)	0.80 (0.42, 1.50)	1.07 (0.82, 1.40)	1.03 (0.72, 1.49)	1.06 (0.72, 1.56)
	>30.00	0.90 (0.70, 1.16)	0.88 (0.66, 1.18)	0.72 (0.32, 1.62)	0.97 (0.73, 1.29)	0.87 (0.60, 1.28)	1.07 (0.66, 1.74)
	Continuous	1.00 (0.99, 1.00)	1.00 (0.99, 1.00)	0.99 (0.97, 1.02)	1.00 (0.99, 1.01)	1.00 (0.99, 1.01)	1.00 (0.99, 1.02)
	Median (>/≤)	0.99 (0.85, 1.16)	1.01 (0.84, 1.21)	0.86 (0.52, 1.40)	1.01 (0.84, 1.23)	1.08 (0.82, 1.41)	0.93 (0.69, 1.26)
Sugar	0.002–0.160	0.85 (0.61, 1.17)	0.87 (0.61, 1.25)	1.13 (0.47, 2.71)	0.90 (0.61, 1.32)	0.76 (0.44, 1.31)	1.15 (0.63, 2.10)
beets^h,i^	>0.160	1.24 (0.80, 1.92)	1.26 (0.78, 2.03)	1.30 (0.49, 3.43)	1.00 (0.66, 1.53)	0.77 (0.42, 1.41)	1.45 (0.90, 2.33)
	Continuous	1.11 (1.04, 1.19)	1.11 (1.02, 1.21)	1.01 (0.78, 1.30)	1.05 (0.94, 1.17)	1.01 (0.85, 1.20)	1.10 (0.99, 1.23)
	Any/None	1.04 (0.75, 1.43)	1.06 (0.75, 1.49)	1.22 (0.58, 2.55)	0.95 (0.69, 1.30)	0.76 (0.48, 1.20)	1.30 (0.84, 2.00)
Wheat	0.10–0.71	1.13 (0.85, 1.51)	0.92 (0.67, 1.26)	2.32 (1.05, 5.12)	1.24 (0.89, 1.73)	1.20 (0.77, 1.86)	0.96 (0.58, 1.60)
	0.72–2.04	0.88 (0.67, 1.17)	0.78 (0.57, 1.08)	1.51 (0.64, 3.55)	1.16 (0.82, 1.63)	1.01 (0.66, 1.53)	1.12 (0.68, 1.84)
	>2.04	0.90 (0.68, 1.19)	0.76 (0.56, 1.04)	1.69 (0.73, 3.94)	1.06 (0.75, 1.49)	0.98 (0.64, 1.48)	1.06 (0.64, 1.76)
	Continuous	0.97 (0.94, 0.99)	0.97 (0.93, 1.00)	0.98 (0.90, 1.08)	0.98 (0.95, 1.02)	0.99 (0.94, 1.03)	0.98 (0.93, 1.03)
	Median (>/≤)	0.81 (0.69, 0.95)	0.83 (0.68, 1.00)	0.83 (0.52, 1.31)	0.94 (0.77, 1.13)	0.89 (0.67, 1.19)	1.08 (0.81, 1.543

^a^Models were restricted to counties with <300,000 people and adjusted for sex, race (white, black, other), year of diagnosis, state of residence at diagnosis, median household income, population density, and education. County was treated as a repeated measure. ^b^Crop density quartiles except for barley, dry beans, sorghum, and sugar beets for which non-zero values were categorized as ≤ median and > median. Continuous RR is per one unit change of crop density. ^c^Indiana was not included in these models. ^d^42.7 % of barley measures were 0. ^e^ Illinois not included in this model. ^f^74.3 % of dry bean measures were 0. ^g^46.9 % of sorghum measures were 0. ^h^Michigan and Ohio only. ^i^79.6 % of sugar beet measures were 0

Spline models revealed a positive non-linear trend in the association of CNS cancers and hay (*p* = 0.021). Spline models could not be fitted for sugar beets or dry beans due to an insufficient number of counties harvesting these crops. No other significant non-linear trends were observed for the other crops.

### Individual state analysis

We stratified models by state to determine if associations by state differed from the overall models. The results from these analyses are in Additional file 1: Tables S1 through S6. Data for many analyses were sparse, which resulted in many models not converging. However, some heterogeneity was observed in the analyses of crop density and cancer incidence among the states.

We found statistically significant positive associations between density of agricultural cropland and total leukemia and ALL in Illinois (fourth vs. first quartile RR_Leukemia_ = 1.98, 95 % CI = 1.19–3.28) (Additional file 1: Table S1) but not for the other states. Illinois counties with greater than the median acreage of corn also had significantly higher rates of leukemias and ALL (RR_Leukemia_ = 2.09, 95 % CI = 1.31–3.32). In contrast, counties with higher density of wheat had lower incidence rates.

No significant linear trends were observed between any of the crop density-cancer incidence combinations in Indiana or Iowa (Additional file 1: Tables S2 and S3). A statistically significant positive association was observed between corn and CNS/PNS incidence (fourth vs. first quartile RR = 2.76, 95 % CI = 1.01–7.49) in Iowa (Additional file 1: Table S3).

In Michigan, we found statistically significant positive associations between density of dry beans, oats, and sugar beets and total leukemias and ALL (RR_Leukemia_ 1 % = 1.01, 95 % CI = 1.00–1.02; RR_Leukemia_ 1 % = 1.19, 95 % CI = 1.03–1.37; RR_Leukemia_ 1 % = 1.01, 95 % CI = 1.01–1.02, respectively) (Additional file 1: Table S4). Based on a limited range, there were also positive associations between wheat density and total leukemia and sorghum density and AML.

The median density of corn acreage in Missouri was lower than the other states and higher densities were associated with lower leukemia incidence rates (Additional file 1: Table S5). In contrast to the positive associations that we observed between dry beans and sugar beets and leukemia in Michigan, dry bean density was associated with lower leukemia rates in Missouri and sugar beet density was associated with lower rates in Ohio (Additional file 1: Table S6).

Unlike the six state analysis, we observed many significant positive associations for CNS and PNS cancers combined, including oats (Illinois, Michigan, and Missouri, Additional file 1: Tables S1, S4, and S5), and sorghum (Michigan, Additional file 1: Table S4). Additionally, a significant positive trend was found for PNS cancers and oats in Illinois (Additional file 1: Table S1) and sugar beets in Ohio (Additional file 1: Table S6). In contrast, in Illinois, sorghum and soybeans were inversely associated with CNS/PNS and PNS incidence rates, respectively. Sorghum density was also inversely associated with CNS/PNS incidence in Missouri and Ohio (Additional file 1: Tables S5 and S6).

## Discussion

In our analysis of childhood cancers and crop density in six Midwestern states, we found statistically significant evidence for a positive exposure-response relationship between crop density of dry beans and incidence of total leukemia and ALL; oats and AML; and sugar beets and total leukemia and ALL. State specific models revealed additional statistically significant associations, but results were inconsistent across states. This may be due to the rarity of crops within certain states or differences in agricultural chemical use between the states. To our knowledge this study is the first to report these associations. These findings could point to the impact of more specific pesticide exposures and childhood cancer risk.

The majority of epidemiologic studies examining pesticide exposure and childhood cancer have evaluated parental occupational exposure. Several studies detected a positive association between parental occupational exposure to pesticides and total childhood cancers [4], childhood leukemia [1–3, 22, 23], and childhood brain cancer [2, 3, 24]. PNS cancers, specifically neuroblastomas, have also shown positive associations with parental occupational exposure to pesticides [2, 3], but a recent meta-analysis of paternal occupational pesticide exposure was negative [25]. Additional studies of farm residence and other farm exposures such as exposure to farm animals and childhood cancer risks showed significant positive associations with brain tumors [26, 27], primitive neuroectodermal tumors (PNET) [28], and positive, but not statistically significant associations with leukemia [29].

Few studies have examined residential exposures to agricultural pesticides and the results from these studies have been mixed. Two studies examined pesticide use in California using the California Pesticide Use Reporting database and childhood cancer incidence rates at the census block group level [30, 31]. These investigators found no associations between agricultural pesticides (individual, chemical groups, or toxicologic groups) and childhood ALL or gliomas, except for a positive significant association between the 90^th^ percentile of propargite, a pesticide used to kill mites, and leukemia (RR = 1.48, 95 % CI = 1.03–2.13) [30]. In a case–control study of childhood leukemia in California, Rull et al. [32] compared the density of applied pesticides within half a mile from the birth residence for 213 cases and 268 controls and found positive associations with pesticides categorized into chlorinated phenols, organophosphates, and triazines. However, these findings were only significant at the middle tertile of exposure and the strength of association weakened as exposure increased.

Carozza et al. [33] compared agricultural land use density around the residence at birth for 1778 childhood cancer cases and 1802 controls in Texas using digital maps created from aerial photographs. Although not statistically significant, measures of association with leukemias, CNS, and PNS cancers were generally higher when using density of agricultural land within a 1000 m buffer of the residence at birth than when using distance of agricultural land from the birth residence. Carozza et al. [6] used Census of Agriculture data from 1997 across 25 U.S. states to assess the impact of agricultural land use density on childhood cancer incidence. Compared to counties with low crop density (<20 %), incidence was significantly increased in counties with high crop density (≥60 %) for leukemia (OR = 1.2, 95 % CI = 1.1–1.3) and CNS cancers (OR = 1.3, 95 % CI = 1.1–1.4), respectively. In analyses by crop type, with the reference group as counties with no acreage of the specified crop and <20 % of agricultural land within the county, significant associations were observed between corn density and sympathetic nervous system tumors (OR = 1.3, 95 % CI = 1.1–1.5), and soybeans and sympathetic nervous system tumors (OR = 1.3, 95 % CI = 1.1–1.6) and AML (OR = 1.4, 95 % CI = 1.1–1.7).

We observed significant positive trends in our analyses of dry beans, oats, and sugar beets crop density in spite of the limited acreage of these crops in the six states. Cantor and Fraumeni [34] reported significantly higher mortality rates for non-Hodgkin’s lymphoma (NHL) mortality across all ages in U.S. counties with sugar beet processing facilities. An ecologic study by Blair et al. [35] found an increase in leukemia mortality among adults working in the sugar and confectionary industry, although sugar beets were not specifically mentioned as being grown in these counties. Of the three crops, for which we observed positive associations with leukemia, only oats were examined in the 25 state analysis by Carozza et al. [6] and no association between oat density and AML incidence was found. We were unable to examine primitive neuroectodermal tumors separately, which they found to be positively associated with oat density. However, we did find significant positive associations between oats and CNS and PNS cancers combined in Illinois, Michigan, and Missouri. We were unable to replicated the positive association between corn and PNS tumors and AML and sympathetic nervous system tumors that were reported by Carozza et al. [6]. We found a significant positive association between soybeans and CNS tumors in Ohio, but an inverse association with CNS/PNS incidence in Illinois.

Differences in results between the study conducted by Carozza et al. [6] and our study may be partially explained by differences in study design and analysis. Carozza et al. used categories of low (<20 %), medium (20–< 60 %), and high (≥60 %) for total agricultural land density and a binary measure for crop-specific analyses (any of specific crop versus none and less than 20 % cropland); whereas we evaluated quartiles, binary, and continuous crop densities. We excluded counties with populations of 300,000 or greater, while Carozza et al. did not use any restrictions based on population size. Our study examined children under the age of five while Carozza et al. examined children under the age of 15. Our studies included different states (six Midwestern states versus 25 U.S. states; only Illinois, Iowa, and Indiana overlapped). Additionally, our study used acres of crops harvested, while Carozza et al. used acres planted.

Crop density was used as a proxy for exposure to agricultural pesticides in our study. However, we did not have county-level information on the proportion of acres treated with pesticides or the specific types of pesticides used. Based on state-level pesticide use data, the crops with the highest densities in our study, corn and soybeans, had the highest proportion of acres treated with pesticides in these six states (96–99 % treated with herbicides) [36]. The most common herbicide used on corn was atrazine and the most common herbicide used on soybeans was glyphosate. We did not find positive associations between the densities of these commonly grown crops and childhood cancer incidence in the combined analysis, but we did observe a suggestive association between higher density of corn and childhood leukemia in Illinois, without a significant trend. Among the crops that demonstrated significant associations with childhood cancers, oats were the most prevalent. The proportion of oat acres treated with herbicides varied greatly by state with as little as 3 % treated in Iowa and as much as 61 % in Michigan [36]. The most commonly used pesticides on oats in the United States in 2005 were the herbicides 2,4-D, glyphosate, and MCPA [36]. Among the crops that were significantly associated with childhood cancer in our analysis, sugar beets had the highest proportion of acreage treated with herbicides (98 %) [36]. Sugar beets also had a very high proportion of acres treated with insecticides (63 %) and fungicides (72 %) [36]. In 2000, desmedipham (94 % of acres treated), triflusulfuron-methyl (83 %), phenmedipham (80 %), clopyralid (74 %), and tetraconazole (55 %) were the most commonly used pesticides on sugar beets [36]. In 1999, approximately 70 % of dry bean acreage was treated with ethyl dipropythiocarbamate (EPTC), 50 % with trifluralin, and 35 % with metolachlor [37]. Of the pesticides mentioned, triflusulfuron-methyl, trifluralin, and metolachlor are listed as group C – possible human carcinogens by the EPA [38].

Our study had several limitations primarily due to its ecologic study design. Individual level exposures, outcomes, and potential confounders, such as smoking, maternal age, or birth weight, were not available in the county level data used in this study. The use of county of residence at the time of diagnosis may result in additional misclassification of exposure since we did not know how long a child, or the parents lived at the residence and if they had resided there during gestation or in the perinatal period. Additionally, the use of crop density as an indicator of potential exposure to agricultural pesticides does not take into account direct routes of exposure or variables that could impact exposure such as parental occupation, time spent outdoors, and home ventilation.

Despite the limitations, this study had several strengths. First, with over 1.6 million children this study had a relatively large size for studying childhood cancers, which are rare. Studying crop density measures across a large area of the Midwestern United States allowed for diversity in the types of crops examined while providing enough overlap in the major crop types to examine associations in pooled analyses. By linking residence county at diagnosis to land use data we were able to eliminate the potential for recall bias, which may have led to differential misclassification of pesticide exposure in many occupational case–control studies [39]. Perhaps the greatest advantage of this approach is that it relies on publically available data and thus is useful for conducting hypothesis generating studies at relatively low cost.

## Conclusions

Using publicly available data sources we have found some evidence for an association between childhood cancer incidence and the production of dry beans, oats, and sugar beets. However, these findings need to be replicated in other studies with detailed information on individual level exposures, pesticide use data, and potentially confounding factors that could not be taken into account in this ecologic study.

## Additional file

Additional file 1:
**Table S1.** Estimated rate ratios (RRs) and 95 % confidence intervals (CI) of childhood cancers (0–4 years of age) associated with cropland density by crop type in Illinois, 2004–2008. Table S2. Estimated rate ratios (RRs) and 95 % confidence intervals (CI) of childhood cancers (0–4 years of age) associated with cropland density by crop type in Indiana, 2004–2008. Table S3. Estimated rate ratios (RRs) and 95 % confidence intervals (CI) of childhood cancers (0–4 years of age) associated with cropland density by crop type in Iowa, 2004–2008. Table S4. Estimated rate ratios (RRs) and 95 % confidence intervals (CI) of childhood cancers (0–4 years of age) associated with cropland density by crop type in Michigan, 2004–2008. Table S5. Estimated rate ratios (RRs) and 95 % confidence intervals (CI) of childhood cancers (0–4 years of age) associated with cropland density by crop type in Missouri, 2004–2008. Table S6. Estimated rate ratios (RRs) and 95 % confidence intervals (CI) of childhood cancers (0–4 years of age) associated with cropland density by crop type in Ohio, 2004–2008. (DOC 428 kb)
