# Supplementary material for: Agricultural crop density and risk of childhood cancer in the midwestern United States: an ecologic study
Source: Environ Health. 2015 Oct 15;14:82. doi: 10.1186/s12940-015-0070-3 (PMC4606898; doi:10.1186/s12940-015-0070-3)
Supplement: Additional file 1: — Table S1. Estimated rate ratios (RRs) and 95 % confidence intervals (CI) of childhood cancers (0–4 years of age) associated with cropland density by crop type in Illinois, 2004–2008. Table S2. Estimated rate ratios (RRs) and 95 % confidence intervals (CI) of childhood cancers (0–4 years of age) associated with cropland density by crop type in Indiana, 2004–2008. Table S3. Estimated rate ratios (RRs) and 95 % confidence intervals (CI) of childhood cancers (0–4 years of age) associated with cropland density by crop type in Iowa, 2004–2008. Table S4. Estimated rate ratios (RRs) and 95 % confidence intervals (CI) of childhood cancers (0–4 years of age) associated with cropland density by crop type in Michigan, 2004–2008. Table S5. Estimated rate ratios (RRs) and 95 % confidence intervals (CI) of childhood cancers (0–4 years of age) associated with cropland density by crop type in Missouri, 2004–2008. Table S6. Estimated rate ratios (RRs) and 95 % confidence intervals (CI) of childhood cancers (0–4 years of age) associated with cropland density by crop type in Ohio, 2004–2008. (DOC 428 kb) [file 12940_2015_70_MOESM1_ESM.doc]

**Additional file 1**

Agricultural Crop Density and Risk of Childhood Cancer in the Midwestern United States: an Ecologic Study

Benjamin J. Booth, Mary H. Ward, Mary E. Turyk, Leslie T. Stayner

**Additional file 1: Table S1.** Estimated rate ratios (RRs) and 95% confidence intervals (CI) of childhood cancers (0-4 years of age) associated with cropland density by crop type in Illinois, 2004-2008.

**Additional file 1: Table S2.** Estimated rate ratios (RRs) and 95% confidence intervals (CI) of childhood cancers (0-4 years of age) associated with cropland density by crop type in Indiana, 2004-2008.

**Additional file 1: Table S3.** Estimated rate ratios (RRs) and 95% confidence intervals (CI) of childhood cancers (0-4 years of age) associated with cropland density by crop type in Iowa, 2004-2008.

**Additional file 1: Table S4.** Estimated rate ratios (RRs) and 95% confidence intervals (CI) of childhood cancers (0-4 years of age) associated with cropland density by crop type in Michigan, 2004-2008.

**Additional file 1: Table S5.** Estimated rate ratios (RRs) and 95% confidence intervals (CI) of childhood cancers (0-4 years of age) associated with cropland density by crop type in Missouri, 2004-2008.

**Additional file 1: Table S6.** Estimated rate ratios (RRs) and 95% confidence intervals (CI) of childhood cancers (0-4 years of age) associated with cropland density by crop type in Ohio, 2004-2008.

**Supplemental Table 1.** Estimated rate ratios (RRs)^a^ and 95% confidence intervals (CI) of childhood cancers (0-4 years of age) associated with cropland density overall and by crop type in Illinois, 2004-2008

| Crop | Crop density (%)^b^ | Total Leukemia | ALL | AML | CNS/PNS | CNS | PNS |
| --- | --- | --- | --- | --- | --- | --- | --- |
| Agricultural | 59.7-72.0 | 0.74 (0.52, 1.07) | 0.72 (0.44, 1.20) | - | 0.60 (0.31, 1.15) | - | 0.68 (0.31, 1.52) |
| Land^c^ | 72.1-78.9 | 1.45 (0.78, 2.70) | 1.41 (0.69, 2.85) | - | 0.96 (0.59, 1.56) | - | 0.59 (0.23, 1.46) |
|  | >79.0 | **1.98 (1.19, 3.28)** | **1.84 (1.01, 3.33)** | - | 0.67 (0.37, 1.21) | - | 1.06 (0.44, 2.53) |
|  | Continuous | 1.01 (0.99, 1.03) | 1.01 (0.99, 1.03) | - | 0.99 (0.98, 1.00) | - | 1.00 (0.98, 1.03) |
|  | Median (>/≤) | **1.97 (1.26, 3.10)** | **1.88 (1.13, 3.13)** | - | 0.99 (0.61, 1.61) | - | 0.99 (0.49, 2.01) |
| Barley^d^ | 0.002-0.009 | 0.84 (0.49, 1.45) | 0.90 (0.52, 1.57) | - | 1.05 (0.58, 1.90) | - | 0.79 (0.28, 2.17) |
|  | >0.009 | **0.62 (0.39, 0.99)** | 0.75, 0.44, 1.26) | - | 1.24 (0.68, 2.29) | - | 0.92 (0.40, 2.12) |
|  | Continuous^c^ | 0.24 (0.02, 3.65) | 0.24 (0.02, 3.65) | - | 2.35 (0.32, 17.24) | - | 4.49 (0.40, 50.49) |
|  | Any/None | 0.74 (0.47, 1.18) | 0.83 (0.51, 1.36) | - | 1.14 (0.70, 1.86) | - | 0.85 (0.40, 1.80) |
| Corn^c^ | 24.7-34.4 | 0.69 (0.33, 1.43) | 0.64 (0.27, 1.50) | - | 0.82 (0.35, 1.91) | - | **3.85 (1.17, 12.68)** |
|  | 34.5-43.3 | **1.85 (1.02, 3.36)** | 1.49 (0.72, 3.08) | - | 1.03 (0.52, 2.05) | - | 1.96 (0.59, 6.54) |
|  | >43.3 | 1.64 (0.84, 3.22) | 1.33 (0.63, 2.77) | - | 0.86 (0.42, 1.75) | - | 2.77 (0.85, 8.99) |
|  | Continuous | 1.02 (0.99, 1.04) | 1.01 (0.99, 1.03) | - | 1.00 (0.98, 1.02) | - | 1.01 (0.99, 1.04) |
|  | Median (>/≤) | **2.09 (1.31, 3.32)** | **1.74 (1.04, 2.89)** | - | 1.03 (0.63, 1.69) | - | 1.03 (0.49, 2.14) |
| Hay^f^ | 0.90-1.56 | 0.94 (0.53, 1.66) | 0.89 (0.48, 1.63) | - | 0.94 (0.50, 1.77) | - | 0.81 (0.30, 2.18) |
|  | 1.57-2.48 | 0.59 (0.33, 1.04) | **0.50 (0.27, 0.94)** | - | 1.53 (0.87, 2.69) | - | 1.71 (0.70, 4.17) |
|  | >2.48 | 0.72 (0.40, 1.32) | 0.82 (0.45, 1.51) | - | 1.32 (0.77, 2.27) | - | 0.79 (0.31, 2.01) |
|  | Continuous | 0.94 (0.85, 1.05) | 0.96 (0.86, 1.08) | - | 1.04 (0.98, 1.11) | - | 1.02 (0.92, 1.14) |
|  | Median (>/≤) | **0.67 (0.46, 0.99)** | 0.69 (0.46, 1.04) | - | 1.48 (0.99, 2.21) | - | 1.45 (0.76, 2.76) |
| Oats^e^ | 0.011-0.037 | 1.15 (0.60, 2.21) | 1.05 (0.52, 2.13) | - | 0.66 (0.30, 1.44) | - | 0.65 (0.21, 1.99) |
|  | 0.038-0.099 | 1.55 (0.95, 2.53) | 1.30 (0.75, 2.25) | - | 1.15 (0.60, 2.20) | - | 0.84 (0.33, 2.12) |
|  | >0.099 | 1.08 (0.77, 1.52) | 0.88 (0.60, 1.31) | - | 1.71 (0.93, 3.13) | - | **2.25 (1.15, 4.39)** |
|  | Continuous | 1.02 (0.91, 1.15) | 1.00 (0.85, 1.18) | **-** | **1.17 (1.03, 1.33)** | **-** | **1.23 (1.09, 1.39)** |
|  | Median (>/≤) | 1.20 (0.81, 1.76) | 1.03 (0.68, 1.56) | - | 1.73 (1.06, 2.81) |  | **1.91 (1.02, 3.60)** |
| Sorghum^e^ | 0.002-0.025 | 0.76 (0.44, 1.32) | 0.75 (0.41, 1.37) | - | 0.84 (0.46, 1.52) | - | 0.69 (0.29, 1.66) |
|  | 0.026-0.174 | 0.84 (0.47, 1.50) | 0.89 (0.43, 1.85) | - | 0.70 (0.38, 1.28) | - | 0.49 (0.17, 1.37) |
|  | >0.174 | **0.45 (0.26, 0.76)** | **0.55 (0.31, 0.96)** | - | **0.46 (0.25, 0.86)** | - | **0.35 (0.16, 0.75)** |
|  | Continuous | 0.95 (0.90, 0.99) | 0.97 (0.93, 1.01) | - | 0.99 (0.91, 1.08) | - | **0.87 (0.78, 0.99)** |
|  | Median (>/≤) | **0.66 (0.46, 0.95)** | 0.76 (0.51, 1.13) | - | **0.60 (0.37, 0.97)** | - | **0.47 (0.24, 0.91)** |
| Soybeans^c^ | 23.0-28.1 | 1.00 (0.68, 1.48) | 0.83 (0.51, 1.35) | - | 0.70 (0.39, 1.25) | - | 0.64 (0.30, 1.39) |
|  | 28.2-32.0 | 1.44 (0.82, 2.54) | 1.38 (0.77, 2.48) | - | **0.58 (0.38, 0.89)** | - | **0.38 (0.15, 0.99)** |
|  | >32.0 | 1.10 (0.65, 1.87) | 1.14 (0.62, 2.11) | - | **0.49 (0.25, 0.96)** | - | 0.79 (0.36, 1.70) |
|  | Continuous | 1.01 (0.98, 1.04) | 1.01 (0.98, 1.05) | - | **0.96 (0.94, 0.98)** | - | 0.97 (0.93, 1.01) |
|  | Median (>/≤) | 1.31 (0.88, 1.95) | 1.43 (0.95, 2.16) | - | 0.67 (0.44, 1.00) | - | 0.70 (0.37, 1.34) |
| Wheat^f^ | 0.43-1.03 | 0.86 (0.57, 1.31) | 1.03 (0.63, 1.68) | - | 0.92 (0.55, 1.56) | - | 1.21 (0.57, 2.57) |
|  | 1.04-2.71 | 0.78 (0.42, 1.46) | 0.97 (0.49, 1.94) | - | 0.93 (0.48, 1.81) | - | 0.62 (0.23, 1.72) |
|  | >2.71 | **0.57 (0.37, 0.86)** | 0.75 (0.45, 1.23) | - | 0.49 (0.22, 1.08) | - | **0.41 (0.16, 1.06)** |
|  | Continuous | **0.96 (0.94, 0.99)** | 0.97 (0.95, 1.00) | - | 0.97 (0.92, 1.03) | - | 0.94 (0.87, 1.02) |
|  | Median (>/≤) | 0.72 (0.47, 1.11) | 0.84 (0.53, 1.33) | - | 0.73 (0.42, 1.27) | - | **0.46 (0.22, 0.96)** |

^a^ Models were restricted to counties with <300,000 people and adjusted for sex, race (white, black, other), year of diagnosis, median household income, population density, and education. County was treated as a repeated measure. ^b^ Crop density quartiles except for barley for which non-zero values were categorized as ≤ median and > median. ^c^ RR per one unit change in crop density. ^d^ 59.5% of barley measures were 0.

^e^ RR per 0.1 unit change in crop density. ^f^ RR per 0.5 unit change in crop density. - Model did not converge.

**Supplemental Table 2.** Estimated rate ratios (RRs)^a^ and 95% confidence intervals (CI) of childhood cancers (0-4 years of age) associated with cropland density overall and by crop type in Indiana, 2004-2008

| Crop | Crop density (%)^b^ | Total Leukemia | ALL | AML | CNS/PNS | CNS | PNS |
| --- | --- | --- | --- | --- | --- | --- | --- |
| Agricultural | 40.3-58.9 | 0.79 (0.45, 1.37) | 0.95 (0.54, 1.69) | - | 0.99 (0.56, 1.72) | - | - |
| Land^c^ | 59.0-72.0 | 0.69 (0.46, 1.04) | 0.77 (0.50, 1.19) | - | 1.12 (0.67, 1.89) | - | - |
|  | >72.0 | 1.15 (0.71, 1.85) | 1.12 (0.66, 1.93) | - | 1.17 (0.67, 2.02) | - | - |
|  | Continuous | 0.99 (0.98, 1.01) | 0.99 (0.99, 1.01) | - | 1.00 (0.99, 1.01) | - | - |
|  | Median (>/≤) | 0.97 (0.67, 1.39) | 0.92 (0.63, 1.36) | - | 1.15 (0.79, 1.68) | - | - |
| Barley^d, e^ | 0.004-0.009 | 0.81 (0.54, 1.22) | 0.89 (0.58, 1.37) |  | 1.04 (0.71, 1.52) | - | - |
|  | >0.009 | 1.05 (0.64, 1.72) | 1.03 (0.63, 1.74) |  | 0.74 (0.45, 1.21) | - | - |
|  | Continuous | 0.86 (0.24, 3.08) | 0.86 (0.24, 3.08) | - | 0.35 (0.08, 1.50) | - | - |
|  | Any/None | 0.93 (0.65, 1.35) | 0.96 (0.65, 1.42) | - | 0.89 (0.62, 1.26) | - | - |
| Beans (dry)^d, f^ | 0.004-0.025 | 0.69 (0.33, 1.44) | 0.71 (0.32, 1.55) | - | 1.15 (0.69, 1.89) | - | - |
|  | >0.025 | 0.75 (0.39, 1.45) | 0.61 (0.31, 1.20) | - | 1.11 (0.63, 1.94) | - | - |
|  | Continuou | 0.75 (0.26, 2.15) | 0.63 (0.17, 2.32) | - | 1.37 (0.72, 2.60) | - | - |
|  | Any/None | 0.72 (0.43, 1.20) | 0.67 (0.38, 1.17) | - | 1.31 (0.75, 1.70) | - | - |
| Corn^c^ | 15.4-26.2 | 0.67 (0.41, 1.10) | 0.88 (0.51, 1.51) | - | 0.78 (0.44, 1.36) | - | - |
|  | 26.3-33.3 | **0.59 (0.37, 0.93)** | 0.70 (0.42, 1.15) | - | 1.04 (0.63, 1.71) | - | - |
|  | >33.3 | 0.96 (0.59, 1.56) | 1.04 (0.61, 1.78) | - | 1.12 (0.64, 1.96) | - | - |
|  | Continuous | 0.99 (0.97, 1.00) | 0.99 (0.97, 1.01) | - | 1.00 (0.98, 1.02) | - | - |
|  | Median (>/≤) | 0.90 (0.64, 1.27) | 0.88 (0.61, 1.25) | - | 1.24 (0.88, 1.76) | - | - |
| Hay^g^ | 1.28-1.88 | 0.80 (0.48, 1.33) | 0.91 (0.52, 1.60) | - | **0.50 (0.31, 0.80)** | - | - |
|  | 1.89-3.65 | 0.70 (0.38, 1.27) | 0.80 (0.42, 1.54) | - | 0.68 (0.40, 1.17) | - | - |
|  | >3.65 | 1.04 (0.67, 1.62) | 1.12 (0.70, 1.79) | - | 0.88 (0.58, 1.32) | - | - |
|  | Continuous | 1.01 (0.96, 1.06) | 1.02 (0.97, 1.07) | - | 1.01 (0.96, 1.07) | - | - |
|  | Median (>/≤) | 0.98 (0.69, 1.41) | 1.01 (0.69, 1.48) | - | 1.09 (0.76, 1.58) | - | - |
| Oats^d^ | 0.006-0.016 | 0.76 (0.52, 1.10) | 0.80 (0.53, 1.20) | - | 1.19 (0.70, 2.04) | - | - |
|  | 0.017-0.044 | 0.79 (0.50, 1.26) | 0.83 (0.50, 1.37) | - | 1.14 (0.62, 2.07) | - | - |
|  | >0.044 | 0.81 (0.49, 1.33) | 0.90 (0.54, 1.50) | - | 0.97 (0.59, 1.59) | - | - |
|  | Continuous | 0.80 (0.63, 1.01) | 0.83 (0.66, 1.04) | - | 0.89 (0.77, 1.02) | - | - |
|  | Median (>/≤) | 0.91 (0.66, 1.25) | 0.96 (0.69, 1.35) | - | 0.95 (0.67, 1.37) | - | - |
| Sorghum^d, h^ | 0.004-0.043 | 0.81 (0.51-1.20) | 0.82 (0.54, 1.25) | - | 1.02 (0.67, 1.56) | - | - |
|  | >0.043 | 0.75 (0.47, 1.18) | 0.77 (0.46, 1.28) | - | 1.28 (0.84, 1.96) | - | - |
|  | Continuous | 1.01 (0.78, 1.32) | 1.03 (0.78, 1.36) | - | 1.12 (0.97, 1.30) | - | - |
|  | Any/None | 0.78 (0.56, 1.10) | 0.80 (0.56, 1.14) | - | 1.13 (0.79, 1.63) | - | - |
| Soybeans^c^ | 14.7-30.0 | 0.61 (0.36, 1.02) | 0.73 (0.42, 1.27) | - | 0.91 (0.54, 1.54) | - | - |
|  | 30.0-31.6 | 0.84 (0.56, 1.27) | 0.93 (0.58, 1.49) | - | 1.09 (0.64, 1.84) | - | - |
|  | >31.6 | 0.90 (0.56, 1.43) | 0.94 (0.58, 1.53) | - | 1.03 (0.60, 1.75) | - | - |
|  | Continuous | 1.00 (0.98, 1.02) | 0.99 (0.98, 1.02) | - | 1.00 (0.98, 1.02) | - | - |
|  | Median (>/≤) | 1.17 (0.85, 1.63) | 1.13 (0.80, 1.61) | - | 1.12 (0.79, 1.60) | - | - |
| Wheat^g^ | 0.72-0.93 | **0.60 (0.37, 0.99)** | 0.62 (0.37, 1.05) | - | 0.55 (0.30, 1.01) | - | - |
|  | 0.94-1.36 | 0.82 (0.50, 1.35) | 0.83 (0.47, 1.47) | - | 1.02 (0.64, 1.64) | - | - |
|  | >1.36 | 0.85 (0.48, 1.48) | 0.89 (0.48, 1.63) | - | 0.85 (0.49, 1.47) | - | - |
|  | Continuous | 0.99 (0.93, 1.05) | 0.98 (0.92, 1.05) | - | 0.99 (0.92, 1.06) | - | - |
|  | Median (>/≤) | 1.15 (0.82, 1.61) | 1.15 (0.81, 1.64) | - | 1.34 (0.94, 1.91) | - | - |

^a^ Models were restricted to counties with <300,000 people and adjusted for sex, race (white, black, other), year of diagnosis, median household income, population density, and education. County was treated as a repeated measure. ^b^ Crop density quartiles except for barley, dry beans, and sorghum for which non-zero values were categorized as ≤ median and > median. ^c^ RR per one unit change of crop density. ^d^ RR per 0.1 unit change in crop density. ^e^ 58.4% of barley measures were 0. ^f^ 84.3% of dry bean measures were 0.

^g^ RR per 0.5 unit change in crop density. ^h^ 56.2% of sorghum measures were 0. - Model did not converge.

**Supplemental Table 3.** Estimated rate ratios (RRs)^a^ and 95% confidence intervals (CI) of childhood cancers (0-4 years of age) associated with cropland density overall and by crop type in Iowa, 2004-2008

| Crop | Crop density (%)^b^ | Total Leukemia | ALL | AML | CNS/PNS | CNS | PNS |
| --- | --- | --- | --- | --- | --- | --- | --- |
| Agricultural | 64.0-76.6 | - | - | - | 0.98 (0.48, 1.99) | - | - |
| Land^c^ | 76.7-86.0 | - | - | - | 1.30 (0.62, 2.73) | - | - |
|  | >86.0 | - | - | - | 1.48 (0.67, 3.25) | - | - |
|  | Continuous | - | - | - | 1.02 (0.99, 1.04) | - | - |
|  | Median (>/≤) | - | - | - | 1.39 (0.77, 2.53) | - | - |
| Barley^d, e^ | 0.003-0.015 | - | - | - | 1.09 (0.61, 1.93) | - | - |
|  | >0.016 | - | - | - | 0.91 (0.44, 1.88) | - | - |
|  | Continuous | - | - | - | 0.49 (0.08, 2.89) | - | - |
|  | Any/None | - | - | - | 1.02 (0.60, 1.72) | - | - |
| Beans (dry)^e, f^ | 0.002-0.027 | - | - | - | 0.89 (0.43, 1.85) | - | - |
|  | >0.027 | - | - | - | 1.36 (0.48, 3.85) | - | - |
|  | Continuous | - | - | - | 1.78 (0.59, 5.37) | - | - |
|  | Any/None | - | - | - | 0.96 (0.50, 1.84) | - | - |
| Corn^c^ | 28.7-37.9 | - | - | - | 2.34 (0.83, 6.55) | - | - |
|  | 38.0-45,2 | - | - | - | 1.31 (0.40, 4.25) | - | - |
|  | >45.2 | **-** | **-** | **-** | **2.76 (1.01, 7.49)** | **-** | **-** |
|  | Continuous | - | - | - | 1.02 (0.99, 1.04) | - | - |
|  | Median (>/≤) | - | - | - | 0.97 (0.56, 1.69) | - | - |
| Hay^g^ | 1.61-2.61 | - | - | - | 0.78 (0.41, 1.51) | - | - |
|  | 2.62-4.74 | - | - | - | 0.70 (0.38, 1.29) | - | - |
|  | >4.74 | - | - | - | 0.45 (0.17, 1.17) | - | - |
|  | Continuous | - | - | - | 0.95 (0.87, 1.04) | - | - |
|  | Median (>/≤) | - | - | - | 0.70 (0.41, 1.18) | - | - |
| Oats^e^ | 0.13-0.21 | - | - | - | 0.88 (0.39, 1.96) | - | - |
|  | 0.22-0.33 | - | - | - | 1.11 (0.47, 2.64) | - | - |
|  | >0.33 | - | - | - | 0.86 (0.42, 1.78) | - | - |
|  | Continuous | - | - | - | 1.00 (0.92, 1.10) | - | - |
|  | Any/None | - | - | - | 1.03 (0.63, 1.68) | - | - |
| Sorghum^e, h^ | 0.001-0.011 | - | - | - | 1.16 (0.61, 2.18) | - | - |
|  | >0.011 | - | - | - | 0.77 (0.31, 1.94) | - | - |
|  | Continuous | - | - | - | 1.01 (0.44, 2.31) | - | - |
|  | Any/None | - | - | - | 1.00 (0.55, 1.82) | - | - |
| Soybeans^c^ | 20.5-26.7 | - | - | - | 1.69 (0.49, 5.84) | - | - |
|  | 26.8-33.2 | - | - | - | 1.75 (0.57, 5.35) | - | - |
|  | >33.2 | - | - | - | 2.68 (0.91, 7.87) | - | - |
|  | Continuous | - | - | - | 1.02 (0.98, 1.07) | - | - |
|  | Median (>/≤) | - | - | - | 1.52 (0.86, 2.67) | - | - |
| Wheat^g^ | 0.01-0.03 | - | - | - | 0.66 (0.33, 1.33) | - | - |
|  | 0.04-0.09 | - | - | - | 0.58 (0.23, 1.49) | - | - |
|  | >0.09 | - | - | - | 0.77 (0.38, 1.55) | - | - |
|  | Continuous | - | - | - | 1.22 (0.47, 3.14) | - | - |
|  | Median (>/≤) | - | - | - | 0.88 (0.52, 1.50) | - | - |

^a^ Models were restricted to counties with <300,000 people and adjusted for sex, race (white, black, other), year of diagnosis, median household income, population density, and education. County was treated as a repeated measure. ^b^ Crop density quartiles except for barley, dry beans, and sorghum for which non-zero values were categorized as ≤ median and > median. ^c^ RR per one unit change of crop density. ^d^ 43.9% of barley measures were 0. ^e^ RR per 0.1 unit change in crop density. ^f^ 70.4% of dry bean measures were 0. ^g^ RR per 0.5 unit change in crop density. ^h^ 64.3% of sorghum measures were 0. - Model did not converge.

**Supplemental Table 4.** Estimated rate ratios (RRs)^a^ and 95% confidence intervals (CI) of childhood cancers (0-4 years of age) associated with cropland density overall and by crop type in Michigan, 2004-2008

| Crop | Crop density (%)^b^ | Total Leukemia | ALL | AML | CNS/PNS | CNS | PNS |
| --- | --- | --- | --- | --- | --- | --- | --- |
| Agricultural | 5.4-16.2 | 1.04 (0.38, 2.79) | 0.62 (0.22, 1.80) | - | 1.19 (0.25, 5.58) | 1.80 (0.38, 8.62) | 0.73 (0.13, 3.99) |
| Land^c^ | 16.3-40.4 | 1.14 (0.42, 3.10) | 1.08 (0.41, 2.80) | - | 1.81 (0.46, 7.08) | 2.49 (0.54, 11.53) | 1.28 (0.28, 5.78) |
|  | >40.4 | 1.58 (0.61, 4.08) | 1.40 (0.54, 3.65) | - | 1.85 (0.49, 7.02) | 1.96 (0.46, 8.37) | 1.83 (0.44, 7.61) |
|  | Continuous | 1.01 (0.99, 1.02) | 1.01 (0.99, 1.02) | 0.99 (0.97, 1.02) | 1.01 (0.99, 1.02) | 1.00 (0.99, 1.02) | 1.01 (0.99, 1.03) |
|  | Median (>/≤) | 1.37 (0.67, 2.84) | 1.71 (0.80, 3.65) | 0.59 (0.10, 3.57) | 1.62 (0.70, 3.79) | 1.41 (0.47, 4.23) | 1.95 (0.77, 4.91) |
| Barley^d^ | 0.006-0.021 | 0.93 (0.58, 1.51) | 1.09 (0.64, 1.83) | 1.33 (0.27, 6.55) | 0.81 (0.43, 1.54) | 1.00 (0.39, 2.61) | 0.61 (0.27, 1.40) |
|  | 0.022-0.040 | 1.08 (0.65, 1.80) | 1.04 (0.58, 1.86) | 2.45 (0.74, 8.16) | 1.23 (0.69, 2.18) | 1.43 (0.55, 3.73) | 1.01 (0.54, 1.90) |
|  | >0.040 | 0.96 (0.43, 2.12) | 1.00 (0.45, 2.21) | 1.01 (0.13, 7.65) | 0.95 (0.40, 2.25) | 0.98 (0.29, 3.28) | 0.90 (0.35, 2.33) |
|  | Continuous | 1.12 (0.68, 1.84) | 1.12 (0.68, 1.84) | 1.10 (0.32, 3.79) | 1.02 (0.61, 1.70) | 1.02 (0.51, 2.02) | 1.02 (0.51, 2.03) |
|  | Median (>/≤) | 1.08 (0.72, 1.62) | 0.97 (0.63, 1.51) | 1.54 (0.57, 4.16) | 1.25 (0.77, 2.05) | 1.25 (0.67, 2.32) | 1.26 (0.70, 2.26) |
| Beans (dry) ^d, e^ | 0.002-0.046 | 1.17 (0.63, 2.17) | 1.10 (0.60, 2.01) | 3.97 (0.37, 42.95) | 0.78 (0.40, 1.55) | 0.86 (0.38, 1.93) | 0.65 (0.29, 1.48) |
|  | 0.047-0.193 | 1.54 (0.93, 2.57) | 1.25 (0.74, 2.12) | 6.27 (0.79, 49.37) | 1.03 (0.57, 1.86) | 0.77 (0.35, 1.70) | 1.47 (0.76, 2.84) |
|  | >0.193 | **2.20 (1.22, 3.95)** | 1.88 (0.95, 3.74) | 6.55 (0.79, 54.55) | 1.17 (0.58, 2.36) | 0.94 (0.35, 2.52) | 1.53 (0.71, 3.31) |
|  | Continuous | **1.01 (1.00, 1.02)** | **1.01 (1.00, 1.02)** | 0.99 (0.97, 1.02) | 1.01 (0.99, 1.01) | 1.01 (0.99, 1.02) | 1.01 (0.99, 1.02) |
|  | Median (>/≤) | **2.15 (1.32, 3.52)** | **1.79 (1.09, 2.96)** | **8.73 (1.13, 67.36)** | 1.15 (0.69, 1.92) | 0.94 (0.51, 1.73) | 1.57 (0.84, 2.96) |
| Corn^c^ | 0.25-1.97 | 1.62 (0.59, 4.46) | 1.73 (0.59, 5.10) | 1.36 (0.08, 22.20) | 3.02 (0.53, 17.13) | 2.74 (0.55, 13.61) | 3.35 (0.43, 26.13) |
|  | 1.98-13.75 | 1.88 (0.60, 5.95) | 1.62 (0.49, 5.38) | 2.70 (0.24, 30.11) | 4.15 (0.78, 22.08) | **5.31 (1.04, 27.23)** | 3.05 (0.48, 19.50) |
|  | >13.75 | 2.03 (0.70, 5.88) | 1.98 (0.63, 6.24) | 1.88 (0.18, 19.45) | 3.21 (0.59, 17.54) | 3.47 (0.69, 17.54) | 3.01 (0.45, 20.05) |
|  | Continuous | 1.01 (0.98, 1.03) | 1.01 (0.99, 1.04) | 0.96 (0.89, 1.04) | 1.00 (0.97, 1.03) | 0.99 (0.95, 1.03) | 1.02 (0.99, 1.05) |
|  | Median (>/≤) | 1.52 (0.73, 3.19) | 1.37 (0.64, 2.95) | 2.08 (0.33, 13.08) | 1.94 (0.84, 4.45) | 2.45 (0.89, 6.78) | 1.48 (0.57, 3.82) |
| Hay^f^ | 1.45-2.88 | 1.13 (0.67, 1.91) | 1.33 (0.65, 2.70) | 0.65 (0.13, 3.21) | 1.50 (0.61, 3.71) | 2.64 (0.81, 8.64) | 0.85 (0.34, 2.14) |
|  | 2.89-3.93 | 1.17 (0.65, 2.10) | 1.21 (0.58, 2.50) | 0.91 (0.22, 3.87) | 1.58 (0.59, 4.18) | 2.50 (0.77, 8.13) | 1.04 (0.40, 2.75) |
|  | >3.93 | 1.06 (0.52, 2.15) | 1.12 (0.49, 2.55) | 1.60 (0.31, 8.24) | 1.72 (0.73, 4.03) | 2.51 (0.87, 7.28) | 1.28 (0.53, 3.09) |
|  | Continuous | 1.01 (0.94, 1.08) | 1.01 (0.94, 1.08) | 1.05 (0.94, 1.18) | 1.05 (0.99, 1.11) | 1.04 (0.97, 1.11) | 1.06 (0.96, 1.17) |
|  | Median (>/≤) | 1.02 (0.67, 1.56) | 0.94 (0.60, 1.46) | 1.59 (0.66, 3.82) | 1.20 (0.75, 1.93) | 1.14 (0.61, 2.13) | 1.28 (0.71, 2.31) |
| Oats^d^ | 0.05-0.14 | 1.13 (0.62, 2.07) | 1.21 (0.61, 2.39) | - | 0.74 (0.f30, 1.81) | 0.52 (0.14, 1.90) | 1.16 (0.41, 3.31) |
|  | 0.15-0.23 | 0.94 (0.50, 1.78) | 1.14 (0.57, 2.28) | - | 1.03 (0.44, 2.43) | 0.91 (0.26, 3.17) | 1.24 (0.45, 3.45) |
|  | >0.23 | 1.75 (0.91, 3.37) | 1.82 (0.88, 3.76) | - | 1.24 (0.52, 2.95) | 1.17 (0.32, 4.23) | 1.34 (0.48, 3.79) |
|  | Continuous | **1.19 (1.03, 1.37)** | **1.16 (1.00, 1.34)** | 1.20 (0.82, 1.76) | **1.18 (1.00, 1.40)** | 1.23 (0.99, 1.51) | 1.13 (0.93, 1.38) |
|  | Median (>/≤) | 1.11 (0.72, 1.72) | 1.19 (0.76, 1.86) | 0.47 (0.15, 1.51) | 1.38 (0.82, 2.31) | 1.59 (0.77, 3.27) | 1.14 (0.66, 1.97) |
| Sorghum^d, g^ | 0.002-0.005 | 1.37 (0.83, 2.26) | 1.38 (0.78, 2.43) | 1.37 (0.37, 5.03) | 0.86 (0.43, 1.70) | 0.87 (0.36, 2.11) | 0.85 (0.39, 1.82) |
|  | >0.005 | 1.01 (0.54, 1.89) | 0.90 (0.46, 1.76) | 1.78 (0.74, 4.30) | **1.75 (1.14, 2.68)** | **2.38 (1.42, 4.00)** | 1.08 (0.60, 1.95) |
|  | Continuous | 1.47 (0.33, 6.68) | 0.86 (0.13, 5.52) | **5.71 (1.00, 32.49)** | **3.66 (1.01, 13.25)** | **6.92 (1.67, 28.75)** | 1.52 (0.31, 7.54) |
|  | Any/None | 1.18 (0.78, 1.78) | 1.13 (0.72, 1.79) | 1.60 (0.71, 3.59) | 1.28 (0.82, 1.99) | 1.56 (0.91, 2.69) | 0.96 (0.57, 1.61) |
| Soybeans^c^ | 0.004-0.436 | 2.15 (0.76, 6.11) | 1.70 (0.58, 4.95) | - | 2.08 (0.48, 9.08) | - | 1.86 (0.32, 10.82) |
|  | 0.437-11.370 | 2.09 (0.64, 6.83) | 1.73 (0.53, 5.60) | - | 2.78 (0.70, 11.00) | - | 2.11 (0.45, 9.83) |
|  | >11.370 | 2.39 (0.79, 7.21) | 1.88 (0.60, 5.87) | - | 2.35 (0.60, 9.23) | - | 2.66 (0.57, 12.42) |
|  | Continuous | 1.00 (0.98, 1.03) | 1.01 (0.98, 1.03) | 0.99 (0.92, 1.07) | 1.00 (0.97, 1.03) | 0.99 (0.95, 1.03) | 1.02 (0.98, 1.06) |
|  | Median (>/≤) | 1.41 (0.68, 2.94) | 1.34 (0.64, 2.81) | 2.49 (0.39, 15.91) | 1.68 (0.71, 3.95) | 1.69 (0.54, 5.22) | 1.66 (0.65, 4.26) |
| Sugar beets^d, h^ | 0.002-0.164 | 1.07 (0.65, 1.75) | 1.17 (0.72, 1.91) | 1.50 (0.36, 6.29) | 0.82 (0.46, 1.45) | 0.65 (0.33, 1.27) | 1.14 (0.56, 2.30) |
|  | >0.164 | 1.67 (0.99, 2.84) | 1.68 (0.94, 3.02) | 1.98 (0.61, 6.38) | 1.11 (0.68, 1.82) | 0.88 (0.44, 1.75) | 1.52 (0.88, 2.60) |
|  | Continuous | **1.01 (1.01, 1.02)** | **1.01 (1.00, 1.02)** | 1.00 (0.98, 1.03) | 1.01 (0.99, 1.02) | 1.00 (0.98, 1.02) | **1.01 (1.00, 1.02)** |
|  | Any/None | 1.39 (0.88, 2.20) | 1.45 (0.91, 2.33) | 1.80 (0.60, 5.35) | 0.98 (0.65, 1.48) | 0.77 (0.45, 1.33) | 1.36 (0.83, 2.22) |
| Wheat^f^ | 0.03-0.34 | 2.36 (0.83, 6.67) | 1.89 (0.68, 5.28) | - | 1.67 (0.45, 6.23) | 2.31 (0.56, 9.49) | 1.00 (0.17, 5.86) |
|  | 0.35-1.60 | 2.46 (0.81, 7.47) | 2.01 (0.67, 5.99) | - | 2.34 (0.68, 8.04) | 2.22 (0.55, 8.95) | 2.54 (0.68, 9.46) |
|  | >1.60 | 2.46 (0.82, 7.37) | 1.99 (0.65, 6.15) | - | 2.07 (0.60, 7.11) | 2.01 (0.54, 7.43) | 2.16 (0.55, 8.46) |
|  | Continuous | **1.04 (1.00, 1.09)** | 1.04 (0.99, 1.07) | 1.03 (0.93, 1.14) | 1.01 (0.96, 1.08) | 1.00 (0.92, 1.08) | 1.04 (0.98, 1.10) |
|  | Median (>/≤) | 1.44 (0.70, 2.96) | 1.37 (0.67, 2.79) | 1.28 (0.16, 10.16) | 1.62 (0.69, 3.78) | 1.24 (0.37, 4.17) | 2.29 (0.86, 6.08) |

^a^ Models were restricted to counties with <300,000 people and adjusted for sex, race (white, black, other), year of diagnosis, median household income, population density, and education. County was treated as a repeated measure. ^b^ Crop density quartiles except for sorghum and sugar beets for which non-zero values were categorized as ≤ median and > median. ^c^ RR per one unit change in crop density. ^d^ RR per 0.1 unit change in crop density. ^e^ 40.3% of dry bean measures were 0, non-zero values were divided into three groups (tertiles of non-zero values). ^f^ RR per 0.5 unit change in crop density. ^g^ 68.8% of sorghum measures were 0. ^h^ 64.9% of sugar beet measures were 0. - Model did not converge.

**Supplemental Table 5.** Estimated rate ratios (RRs)^a^ and 95% confidence intervals (CI) of childhood cancers (0-4 years of age) associated with cropland density overall and by crop type in Missouri, 2004-2008

| Crop | Crop density (%)^b^ | Total Leukemia | ALL | AML | CNS/PNS | CNS | PNS |
| --- | --- | --- | --- | --- | --- | --- | --- |
| Agricultural | 26.2-43.0 | 1.01 (0.64, 1.62) | 1.14 (0.62, 2.08) | - | - | - | - |
| Land^c^ | 43.1-56.8 | 0.64 (0.37, 1.08) | 0.70 (0.35, 1.42) | - | - | - | - |
|  | >56.9 | 0.80 (0.40, 1.60) | 1.05 (0.49, 2.26) | - | - | - | - |
|  | Continuous | 0.99 (0.98, 1.00) | 0.99 (0.98, 1.01) | - | - | - | - |
|  | Median (>/≤) | 0.69 (0.45, 1.07) | 0.77 (0.47, 1.24) | - | - | - | - |
| Barley^d, e^ | 0.002-0.013 | 0.69 (0.47, 1.01) | 0.77 (0.47, 1.28) | - | - | - | - |
|  | >0.013 | 0.93 (0.60, 1.44) | 0.94 (0.56, 1.58) | - | - | - | - |
|  | Continuous | 0.66 (0.34, 1.32) | 0.66 (0.34, 1.32) | - | - | - | - |
|  | Any/None | 0.81 (0.57, 1.15) | 0.86 (0.55, 1.34) | - | - | - | - |
| Beans (dry)^d, f^ | 0.003-0.036 | - | - | - | - | - | - |
|  | >0.036 | - | - | - | - | - | - |
|  | Continuous | **0.05 (0.01, 0.84)** | **0.01 (0.01, 0.14)** | - | - | - | - |
|  | Any/None | 0.67 (0.42, 1.09) | **0.52 (0.36, 0.75)** | - | - | - | - |
| Corn^c^ | 0.40-4.64 | 0.88 (0.55, 1.40) | 0.86 (0.50, 1.48) | - | - | - | - |
|  | 4.65-10.82 | **0.60 (0.36, 0.99)** | **0.51 (0.29, 0.91)** | - | - | - | - |
|  | >10.82 | 0.54 (0.28, 1.04) | 0.56 (0.26, 1.19) | - | - | - | - |
|  | Continuous | **0.96 (0.93, 0.99)** | 0.97 (0.93, 1.01) | - | - | - | - |
|  | Median (>/≤) | **0.61 (0.39, 0.96)** | **0.58 (0.35, 0.95)** | - | - | - | - |
| Hay^g^ | 5.98-9.22 | 0.91 (0.56, 1.50) | 0.91 (0.46, 1.81) | - | - | - | - |
|  | 9.23-12.70 | 1.50 (0.85, 2.65) | 1.20 (0.62, 2.34) | - | - | - | - |
|  | >12.70 | 1.08 (0.66, 1.78) | 1.01 (0.57, 1.78) | - | - | - | - |
|  | Continuous | 1.01 (0.99, 1.03) | 1.01 (0.99, 1.03) | - | - | - | - |
|  | Median (>/≤) | 1.32 (0.91, 1.91) | 1.15 (0.74, 1.78) | - | - | - | - |
| Oats^d^ | 0.01-0.02 | 0.71 (0.41, 1.24) | 0.71 (0.38, 1.33) | - | - | - | - |
|  | 0.03-0.07 | 0.75 (0.50, 1.12) | 0.80 (0.48, 1.35) | - | - | - | - |
|  | >0.07 | 0.78 (0.40, 1.54) | 0.97 (0.48, 1.93) | - | - | - | - |
|  | Continuous | 0.86 (0.48, 1.55) | 1.03 (0.59, 1.80) | **-** | **1.18 (1.00, 1.40)** | **-** | **-** |
|  | Median (>/≤) | 0.89 (0.63, 1.25) | 1.00 (0.67, 1.51) | - | - | - | - |
| Sorghum^d^ | 0.03-0.11 | 0.72 (0.41, 1.27) | 0.85 (0.42, 1.69) | - | - | - | - |
|  | 0.12-0.34 | 1.04 (0.53, 2.02) | 1.10 (0.47, 2.58) | - | - | - | - |
|  | >0.34 | 0.65 (0.40, 1.06) | 0.69 (0.35, 1.35) | - | - | - | - |
|  | Continuous | 0.97 (0.94, 1.01) | 0.97 (0.92, 1.02) | - | - | - | - |
|  | Median (>/≤) | 1.00 (0.68, 1.46) | 0.97 (0.62, 1.54) | - | - | - | - |
| Soybeans^c^ | 0.6-9.4 | 1.05 (0.64, 1.71) | 0.87 (0.48, 1.58) | - | 0.98 (0.62, 1.54) | - | - |
|  | 9.5-19.1 | 0.87 (0.56, 1.33) | 0.75 (0.44, 1.30) | - | 1.30 (0.79, 2.15) | - | - |
|  | >19.1 | 0.55 (0.30, 1.02) | 0.61 (0.29, 1.27) | - | 1.08 (0.60, 1.95) | - | - |
|  | Continuous | 0.98 (0.96, 1.00) | 0.98 (0.96, 1.01) | - | - | - | - |
|  | Median (>/≤) | 0.72 (0.49, 1.06) | 0.74 (0.46, 1.20) | - | - | - | - |
| Wheat^g^ | 0.17-0.96 | 0.93 (0.53, 1.62) | 0.77 (0.39, 1.51) | - | - | - | - |
|  | 0.97-2.71 | 0.85 (0.52, 1.40) | 0.77 (0.41, 1.45) | - | - | - | - |
|  | >2.71 | **0.49 (0.26, 0.91)** | 0.56 (0.30, 1.05) | - | - | - | - |
|  | Continuous | 0.97 (0.93, 1.01) | 0.98 (0.94, 1.03) | - | - | - | - |
|  | Median (>/≤) | 0.72 (0.49, 1.04) | 0.79 (0.50, 1.24) | - | - | - | - |

^a^ Models were restricted to counties with <300,000 people and adjusted for sex, race (white, black, other), year of diagnosis, median household income, population density, and education. County was treated as a repeated measure. ^b^ Crop density quartiles except for barley and dry beans for which non-zero values were categorized as ≤ median and > median. ^c^ RR per one unit change in crop density. ^d^ RR per 0.1 unit change in crop density. ^e^ 50.4% of barley measures were 0. ^f^ 91.9% of dry bean measures were 0. ^g^ RR per 0.5 unit change in crop density. - Model did not converge.

**Supplemental Table 6.** Estimated rate ratios (RRs)^a^ and 95% confidence intervals (CI) of childhood cancers (0-4 years of age) associated with cropland density overall and by crop type in Ohio, 2004-2008

| Crop | Crop density (%)^b^ | Total Leukemia | ALL | AML | CNS/PNS | CNS | PNS |
| --- | --- | --- | --- | --- | --- | --- | --- |
| Agricultural | 19.8-43.5 | **0.72 (0.53, 0.99)** | 0.67 (0.44, 1.03) | - | 1.21 (0.76, 1.92) | 1.13 (0.65, 1.96) | 1.40 (0.64, 3.07) |
| Land^c^ | 43.6-68.9 | 0.70 (0.46, 1.06) | 0.64 (0.36, 1.12) | - | 1.37 (0.80, 2.32) | 1.19 (0.63, 2.27) | 1.75 (0.71, 4.30) |
|  | >69.0 | 0.68 (0.40, 1.14) | 0.48 (0.23, 1.02) | - | 1.34 (0.72, 2.50) | 1.63 (0.82, 3.26) | 0.69 (0.18, 2.70) |
|  | Continuous | 0.99 (0.99, 1.00) | 0.99 (0.98, 1.00) | - | 1.00 (0.99, 1.01) | 1.01 (0.99, 1.02) | 1.00 (0.99, 1.01) |
|  | Median (>/≤) | 0.90 (0.66, 1.23) | 0.81 (0.54, 1.22) | - | 1.17 (0.85, 1.60) | 1.19 (0.78, 1.83) | 1.11 (0.68, 1.80) |
| Barley^d^ | 0.003-0.008 | 0.77 (0.49, 1.19) | 0.70 (0.39, 1.26) | - | 1.17 (0.77, 1.79) | 1.02 (0.64, 1.63) | 1.67 (0.67, 4.16) |
|  | 0.009-0.025 | 0.69 (0.47, 1.01) | 0.75 (0.48, 1.18) | - | 0.84 (0.53, 1.31) | 0.58 (0.33, 1.00) | 1.78 (0.85, 3.72) |
|  | >0.025 | **0.74 (0.57, 0.96)** | **0.60 (0.42, 0.86)** | - | 0.89 (0.66, 1.20) | **0.64 (0.42, 0.98)** | 1.76 (0.96, 3.25) |
|  | Continuous | **0.76 (0.59, 0.98)** | **0.76 (0.59, 0.98)** | - | 0.86 (0.64, 1.16) | 0.69 (0.28, 1.73) | 1.18 (0.73, 1.91) |
|  | Median (>/≤) | 0.81 (0.64, 1.02) | 0.76 (0.56, 1.04) | - | 0.80 (0.61, 1.05) | **0.61 (0.43, 0.85)** | 1.36 (0.85, 2.17) |
| Beans (dry)^d, e^ | 0.003-0.035 | 1.00 (0.62, 1.61) | 1.18 (0.66, 2.12) | - | 0.82 (0.49, 1.37) | 0.72 (0.27, 1.95) | 1.04 (0.59, 1.83) |
|  | >0.035 | 1.01 (0.74, 1.36) | 1.00 (0.66, 1.52) | - | 0.79 (0.45, 1.39) | 0.75 (0.42, 1.37) | 0.86 (0.41, 1.82) |
|  | Continuous | 1.15 (0.71, 1.87) | 1.05 (0.57, 1.95) | - | 0.54 (0.23, 1.28) | 0.47 (0.18, 1.22) | 0.69 (0.23, 2.05) |
|  | Any/None | 1.01 (0.76, 1.33) | 1.08 (0.73, 1.59) | - | 0.81 (0.54, 1.20) | 0.74 (0.42, 1.31) | 0.93 (0.56, 1.54) |
| Corn^c^ | 3.13-11.19 | **0.74 (0.56, 0.98)** | **0.63 (0.43, 0.91)** | - | 1.28 (0.81, 2.03) | 1.27 (0.71, 2.29) | 1.47 (0.66, 3.26) |
|  | 11.20-22.85 | **0.60 (0.39, 0.93)** | **0.53 (0.29, 0.96)** | - | 1.29 (0.78, 2.15) | 0.99 (0.51, 1.93) | 2.13 (0.82, 5.51) |
|  | >22.85 | 0.79 (0.49, 1.25) | 0.61 (0.34, 1.09) | - | 1.62 (0.91, 2.87) | 1.81 (0.94, 3.49) | 1.32 (0.43, 4.02) |
|  | Continuous | 1.00 (0.98, 1.01) | 0.99 (0.97, 1.02) | - | 1.01 (0.99, 1.03) | 1.02 (0.99, 1.04) | 1.00 (0.97, 1.03) |
|  | Median (>/≤) | 0.85 (0.63, 1.16) | 0.80 (0.53, 1.19) | - | 1.18 (0.86, 1.62) | 1.11 (0.72, 1.70) | 1.30 (0.82, 2.08) |
| Hay^f^ | 2.34-3.54 | **1.87 (1.14, 3.05)** | **2.10 (1.11, 3.98)** | - | 1.36 (0.89, 2.09) | 1.47 (0.92, 2.34) | 1.24 (0.58, 2.65) |
|  | 3.55-6.15 | **1.60 (1.02, 2.52)** | 1.44 (0.74, 2.80) | - | 0.93 (0.59, 1.45) | 0.88 (0.51, 1.51) | 1.07 (0.51, 2.25) |
|  | >6.15 | 1.26 (0.73, 2.19) | 1.44 (0.72, 2.85) | - | 0.72 (0.41, 1.27) | 0.58 (0.28, 1.20) | 1.08 (0.45, 2.60) |
|  | Continuous | 1.01 (0.98, 1.04) | 1.01 (0.97, 1.05) | - | 0.97 (0.94, 1.01) | 0.96 (0.91, 1.01) | 1.00 (0.95, 1.05) |
|  | Median (>/≤) | 1.02 (0.77, 1.34) | 0.91 (0.63, 1.32) | - | **0.72 (0.54, 0.96)** | **0.62 (0.41, 0.94)** | 0.95 (0.61, 1.49) |
| Oats^d^ | 0.04-0.09 | 1.18 (0.82, 1.70) | 1.19 (0.78, 1.81) | - | 1.04 (0.71, 1.52) | 1.20 (0.76, 1.89) | 0.77 (0.38, 1.54) |
|  | 0.10-0.21 | 0.83 (0.56, 1.24) | 0.72 (0.43, 1.22) | - | 0.84 (0.57, 1.23) | 0.95 (0.60, 1.51) | 0.63 (0.33, 1.21) |
|  | >0.21 | 0.74 (0.55, 1.01) | **0.59 (0.42, 0.81)** | - | 0.75 (0.55, 1.02) | 0.60 (0.36, 1.02) | 1.00 (0.65, 1.54) |
|  | Continuous | 0.97 (0.94, 1.00) | **0.93 (0.86, 0.99)** | - | 0.96 (0.93, 1.01) | 0.94 (0.84, 1.05) | 1.00 (0.94, 1.06) |
|  | Median (>/≤) | **0.72 (0.56, 0.94)** | **0.60 (0.43, 0.83)** | - | 0.77 (0.60, 1.00) | **0.69 (0.49, 0.99)** | 0.94 (062, 1.43) |
| Sorghum^d, g^ | 0.003-0.020 | 0.88 (0.67, 1.16) | 0.80 (0.56, 1.14) | - | 0.86 (0.59, 1.25) | 0.81 (0.47, 1.38) | 0.98 (0.63, 1.53) |
|  | >0.020 | 1.17 (0.81, 1.68) | 1.08 (0.66, 1.78) | - | 0.70 (0.47, 1.02) | 0.66 (0.43, 1.00) | 0.74 (0.36, 1.50) |
|  | Continuous | 1.18 (0.59, 2.36) | 0.99 (0.44, 2.19) | - | **0.56 (0.33, 0.95)** | 0.49 (0.23, 1.02) | 0.65 (0.18, 2.42) |
|  | Any/None | 1.00 (0.74, 1.33) | 0.91 (0.62, 1.33) | - | 0.79 (0.58, 1.07) | 0.74 (0.49, 1.11) | 0.88 (0.56, 1.39) |
| Soybeans^c^ | 3.1-15.3 | 0.77 (0.56, 1.05) | **0.63 (0.42, 0.93)** | - | 2.34 (0.52, 10.53) | 0.93 (0.52, 1.69) | 1.08 (0.55, 2.11) |
|  | 15.4-32.6 | 0.76 (0.52, 1.11) | 0.63 (0.39, 1.01) | - | 3.44 (0.80, 14.79) | 1.37 (0.76, 2.45) | 1.24 (0.57, 2.68) |
|  | >32.6 | 0.74 (0.44, 1.24) | 0.53 (0.25, 1.14) | - | 2.21 (0.54, 9.09) | 1.31 (0.72, 2.38) | 0.66 (0.21, 2.06) |
|  | Continuous | 0.99 (0.98, 1.00) | 0.99 (0.97, 1.00) | - | 1.01 (0.99, 1.02) | **1.01 (1.00, 1.03)** | 1.00 (0.98, 1.02) |
|  | Median (>/≤) | 0.92 (0.70, 1.21) | 0.86 (0.59, 1.23) | - | 1.26 (0.95, 1.68) | 1.42 (0.96, 2.11) | 1.03 (0.67, 1.59) |
| Sugar beets^d, h^ | 0.03-0.07 | - | - | - | 0.85 (0.26, 2.78) | 1.34 (0.43, 4.18) | - |
|  | >0.07 | - | - | - | 1.35 (0.98, 1.87) | 0.34 (0.07, 1.66) | - |
|  | Continuous | **0.26 (0.10, 0.64)** | **0.20 (0.09, 0.43)** | - | 1.14 (0.99, 1.30) | 0.80 (0.47, 1.34) | **1.49 (1.26, 1.76)** |
|  | Any/None | **0.35 (0.14, 0.87)** | **0.30 (0.12, 0.78)** | - | 1.10 (0.62, 1.97) | 0.83 (0.30, 2.24) | 1.66 (0.62, 4.46) |
| Wheat^f^ | 0.34-2.25 | 0.90 (0.66, 1.24) | 0.85 (0.57, 1.27) | - | 1.27 (0.88, 1.83) | 1.34 (0.80, 2.22) | 1.19 (0.62, 2.28) |
|  | 2.26-5.10 | 0.80 (0.58, 1.10) | 0.66 (0.44, 1.00) | - | 1.16 (0.75, 1.77) | 1.00 (0.56, 1.77) | 1.48 (0.71, 3.06) |
|  | >5.10 | 0.66 (0.42, 1.03) | **0.49 (0.25, 0.97)** | - | 1.24 (0.71, 2.17) | 1.43 (0.77, 2.64) | 0.86 (0.29, 2.52) |
|  | Continuous | 0.98 (0.95, 1.01) | 0.97 (0.93, 1.01) | - | 1.00 (0.97, 1.03) | 1.01 (0.98, 1.04) | 0.98 (0.94, 1.03) |
|  | Median (>/≤) | 0.80 (0.61, 1.05) | **0.67 (0.47, 0.96)** | - | 1.00 (0.74, 1.35) | 0.94 (0.63, 1.40) | 1.12 (0.72, 1.72) |

^a^ Models were restricted to counties with <300,000 people and adjusted for sex, race (white, black, other), year of diagnosis, median household income, population density, and education. County was treated as a repeated measure. ^b^ Crop density quartiles except for dry beans and sorghum for which non-zero values were categorized as ≤ median and > median. ^c^ RR per one unit change in crop density. ^d^ RR per 0.1 unit change in crop density. ^e^ 76.2% of dry bean measures were 0. ^f^ RR per 0.5 unit change in crop density. ^g^ 71.2% of sorghum measures were 0. ^h^ 93.7% of sugar beet measures were 0. - Model did not converge.
